# Discovery of Novel Resistance Mechanisms of *Vibrio parahaemolyticus* Biofilm against Aminoglycoside Antibiotics

**DOI:** 10.3390/antibiotics12040638

**Published:** 2023-03-24

**Authors:** Cuifang Tian, Mengqi Yuan, Qian Tao, Tianming Xu, Jing Liu, Zhenhua Huang, Qian Wu, Yingjie Pan, Yong Zhao, Zhaohuan Zhang

**Affiliations:** 1 College of Food Science and Technology, Shanghai Ocean University, 999# Hu Cheng Huan Road, Shanghai 201306, China; 2 Laboratory of Quality & Safety Risk Assessment for Aquatic Products on Storage and Preservation (Shanghai), Ministry of Agriculture and Rural Affairs, 999# Hu Cheng Huan Road, Shanghai 201306, China; 3 Shanghai Engineering Research Center of Aquatic-Product Processing & Preservation, 999# Hu Cheng Huan Road, Shanghai 201306, China

**Keywords:** biofilm, aminoglycoside antibiotics, antibiotic resistance mechanism, *Vibrio parahaemolyticus*

## Abstract

Inappropriate use of antibiotics eventually leads to the emergence of antibiotic-resistant strains and invalidates the treatment of infectious diseases. Aminoglycoside antibiotics (AGAs) are a class of broad-spectrum cationic antibiotics widely used for the treatment of Gram-negative bacterial infections. Understanding the AGA resistance mechanism of bacteria would increase the efficacy of treating these infections. This study demonstrates a significant correlation between AGA resistance and the adaptation of biofilms by *Vibrio parahaemolyticus* (VP). These adaptations were the result of challenges against the aminoglycosides (amikacin and gentamicin). Confocal laser scanning microscope (CLSM) analysis revealed an enclosure type mechanism where the biological volume (BV) and average thickness (AT) of *V. parahaemolyticus* biofilm were significantly positively correlated with amikacin resistance (BIC) (*p* < 0.01). A neutralization type mechanism was mediated by anionic extracellular polymeric substances (EPSs). The biofilm minimum inhibitory concentrations of amikacin and gentamicin were reduced from 32 µg/mL to 16 µg/mL and from 16 µg/mL to 4 µg/mL, respectively, after anionic EPS treatment with DNase I and proteinase K. Here, anionic EPSs bind cationic AGAs to develop antibiotic resistance. Transcriptomic sequencing revealed a regulatory type mechanism, where antibiotic resistance associated genes were significantly upregulated in biofilm producing *V. parahaemolyticus* when compared with planktonic cells. The three mechanistic strategies of developing resistance demonstrate that selective and judicious use of new antibiotics are needed to win the battle against infectious disease.

## 1. Introduction

Foodborne diseases caused by foodborne pathogens contribute to 420,000 deaths annually [1] and antibiotics are the main treatment agents for treating foodborne infections [2]. The discovery of antibiotics has provided a powerful technical means for humans to resist infection by pathogenic bacteria [1]. However, the overuse and inappropriate application of antibiotics has led to the rapid emergence of drug-resistant or multidrug-resistant pathogenic bacteria [3,4,5]. An estimated 33,110 people die each year in the European Union from antibiotic-resistant infections [6]. Aminoglycoside antibiotics (AGAs) are cationic antibiotics that target bacterial ribosomes and disrupt protein synthesis. AGAs are commonly used to treat severe Gram-negative infections, however, bacterial resistance to AGAs is becoming increasingly common and is leading to poorer treatment outcomes. Research has shown that biofilm formation, antibiotic resistance patterns, and gene expression of *Escherichia coli* are significantly altered when exposed to aminoglycosides [7]. Bacterial resistance to AGAs is usually caused by the modification of aminoglycoside-modifying enzymes, the overexpression of active efflux pump genes, and the methylation of 16S rRNA ribosomal subunits, which reduce the concentration of antibiotics within the bacteria and modify the structure of AGAs [8].

Biofilms are a complex community of microbial cells and extracellular polymeric substances (EPSs) that are composed of exopolysaccharides, eDNA, proteins, lipids, and other biomolecules [9]. Bacteria are able to develop antibiotic resistance by forming biofilms, and the mechanism of this process is complex and multifaceted [10]. About 99% of microorganisms have the ability to form biofilms to ensure survival in adverse environments [11,12]. Planktonic cells that exist in biofilms can become 10–1000 times more resistant to the effects of antimicrobial agents, significantly reducing their sensitivity to antibiotics. [13]. Research has shown that planktonic *Salmonella* decreased by 5–6 Log CFU/mL after 8 h of antibiotic treatment, while the number of bacteria in biofilm only decreased by less than 1 Log CFU/mL [14]. Shenkutie et al. demonstrated that the minimum biofilm eradication concentrations were 44, 407, and 364 times higher than the minimum bactericidal concentrations for colistin, ciprofloxacin, and imipenem, respectively [15]. Goodyear et al. obtained similar results for *Pseudomonas aeruginosa* biofilms [16].

*V. parahaemolyticus* is a foodborne pathogen that is known to form biofilms, which can increase bacterial resistance and contribute to foodborne disease burdens [17,18,19,20]. There exists *V. parahaemolyticus* isolates that are 90.5% antibiotic resistant to amikacin [21,22]. *V. parahaemolyticus* cells that grow in biofilms are more resilient to environmental changes and can acquire stronger antibiotic resistance through mutation or by accepting resistance plasmids from other species [23]. Despite this, there is still a lack of research on the antibiotic resistance mechanisms of *V. parahaemolyticus* biofilms to AGAs.

We conducted an experiment to compare the antibiotic resistance of *Vibrio parahaemolyticus* plankton cells and cells that were growing in biofilms. This study aimed to reveal novel resistance mechanisms of a *V. parahaemolyticus* biofilm against AGAs and to provide researchers with tools for designing effective therapies against this pathogen.

## 2. Results

### 2.1. No Linear Correlation between Antibiotic Resistance of Planktonic Cells and Biofilm-Forming Ability

The biofilm biomass of 32 *V. parahaemolyticus* strains was measured using crystal violet staining and this biomass measurement was used as a proxy for biofilm forming ability. According to this grading standard, the ability of the *V. parahaemolyticus* strains to form biofilm are shown in Table 1. A total of 29 *V. parahaemolyticus* strains had acceptable biofilm formation ability, while the remaining three food-derived strains (VPD8, VPR106, and VPR111) had a limited ability to form biofilms. The clinical strains such as VPC17, VPC20, and VPC21 were prolific biofilm producers when compared to the other food-derived strains such as VPD14, VPD33, VPD34, etc. Once the biofilm biomass measurements were completed, minimum inhibitory concentrations (MIC) for the 32 *V. parahaemolyticus* planktonic strains were determined in the presence of eight antibiotics. A microdilution broth method was used to measure the MICs (Table 2).

The statistical software SPSS (version 25) was used to analyze the correlation between the biofilm-forming ability of *V. parahaemolyticus* and MICs of the eight antibiotics. The correlation was measured using the Spearman correlation coefficient (SCC) and there was no linear relationship between the antibiotic resistance of planktonic cells and biofilm-forming ability (*p* > 0.05) (Table 3).

### 2.2. There Is a Significant Correlation between Antibiotic Resistance of Cells Growing in Biofilms and Biofilm-Forming Ability

Using an inoculation needle method, 29 strains with biofilm-forming ability were challenged with eight antibiotics and their biofilm minimum inhibitory concentration (BIC) against these antibiotics were measured (Table 4). Note that the biofilm forming ability of the strains did not change.

SPSS was again used to analyze the biofilm formation ability of *V. parahaemolyticus* and the BIC, and the Spearman correlation coefficient was used to quantify their correlation. The aminoglycoside amikacin BIC for *V. parahaemolyticus* showed a significant linear correlation to the ability to form biofilms (*p* < 0.01), while there was no significant correlation for the remaining antibiotics (Table 5).

### 2.3. Biofilms Are More Protective against Antibiotics

**Figure 1:**
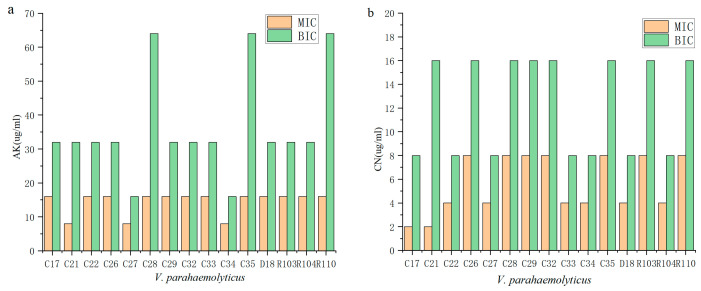
The MIC and BIC of *V. parahaemolyticus* against aminoglycoside antibiotics (C16–C35 refer to VPC16–VPC35. These strains were clinical isolates, which were from clinical diarrhea samples; D18 refers to VPD18, R103–R110 refers to VPR103–VPR110, which are from aquatic products and carry the *tdh* and *trh* virulence genes, respectively). (**a**) Amikacin. (**b**) Gentamicin.

We compared the antibiotic resistance of planktonic cells with cells growing in biofilms (Figure 1). The antibiotic resistance of cells growing in biofilm to amikacin was stronger when compared to planktonic cells, and the resistance of 21 strains to gentamicin increased (Figure 1b). The antibiotic resistance of VPC21 to two antibiotics changed significantly where against AK, the planktonic cells exhibited a MIC of 8 µg/mL and the biofilm cells exhibited a BIC of 32 µg/mL. Against CN, these values were 2 µg/mL and 16 µg/mL (Figure 1).

### 2.4. Biofilm Enhances Antibiotic Resistance through Its Own Structural Characteristics

**Figure 2:**
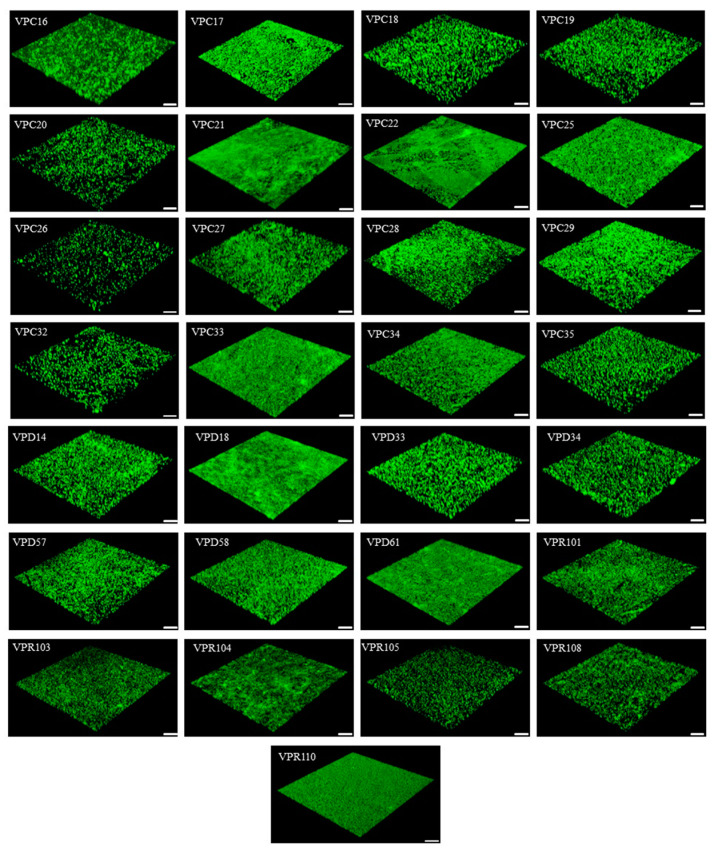
Representative 3D CLSM images of the biofilms formed (VPC16-VPC35 are clinical isolates from clinical diarrhea samples; VPD14-VPD61 and VPR103-VPR110 are from aquatic products and carry the *tdh* and *trh* virulence genes respectively; Scale: 25 μm; 40×).

This study concluded that the antibiotic resistance of the biofilm cells to AGAs was significantly stronger than that of the planktonic cells. One of the factors related to this observation could be the biofilm structure. The structural characteristics of biofilms such as biological volume, average thickness, and biofilm roughness are closely related to the diversity of biofilms [24]. The biofilm of 29 *V. parahaemolyticus* strains with biofilm-forming ability were observed by CLSM, and representative 3D images are shown in Figure 2. The biofilm structure parameters of the strains (biovolume, average thickness, biofilm roughness) were derived using the ISA-2 software (provided by prof. Haluk Beyenal, Montana State University) (Table 6). The results showed that the biofilm biomass of the clinical isolates was generally higher than that of the food isolates, but the average thickness and roughness of the biofilm did not differ significantly.

SPSS was used to analyze the relationship between BIC and the structural parameters of the biofilms, then the correlations were quantified using the Spearman correlation coefficient. The biomass of *V. parahaemolyticus* was significantly correlated with the BIC of levofloxacin and gentamicin (*p* < 0.05), and more significantly with amikacin (*p* < 0.01) (Table 7). Additionally, the average thickness of the *V. parahaemolyticus* biofilm was significantly correlated with the BIC for amikacin (*p* < 0.05) (Table 7). However, there was no correlation between the biofilm roughness and the BIC values of the eight antibiotics. The biofilm biomass and average thickness thus provide protection against antibiotics.

### 2.5. Enzyme-Treated Biofilms Are Less Resistant to Antibiotics

**Figure 3:**
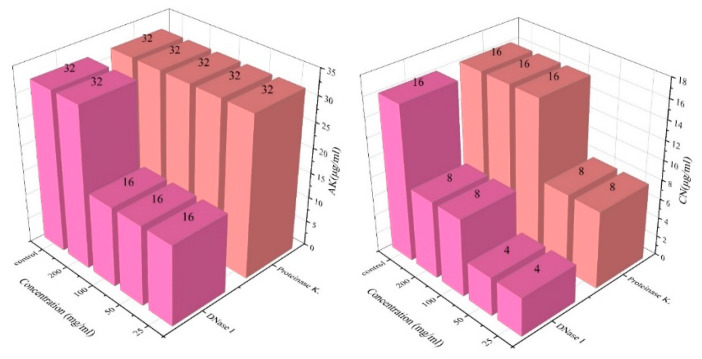
Changes in the BIC of *V. parahaemolyticus* treated with enzymes.

Anions in the EPSs of biofilms (eDNA, exopolysaccharides, and extracellular proteins, etc.) can chelate cations in antibiotics and inhibit the efficacy of antibiotics [25]. We chose VPC21, which possesses a strong biofilm-forming ability, as a test case and analyzed the antibiotic resistance of this strain to AGAs. The BIC changes of VPC21 to amikacin and gentamicin after enzyme treatment are shown in Figure 3. It was found that the BIC of amikacin and gentamicin were reduced to 16 µg/mL and 4 µg/mL after the treatment with DNase I and proteinase K at different concentrations, respectively, indicating that the antibiotic resistance of cells in biofilms were weakened (Figure 3). The results showed that the EPSs in biofilm enhanced the resistance of *V. parahaemolyticus* to AGAs.

### 2.6. There Were Significant Differences in Gene Expression between Biofilm and Planktonic Cells

**Figure 4:**
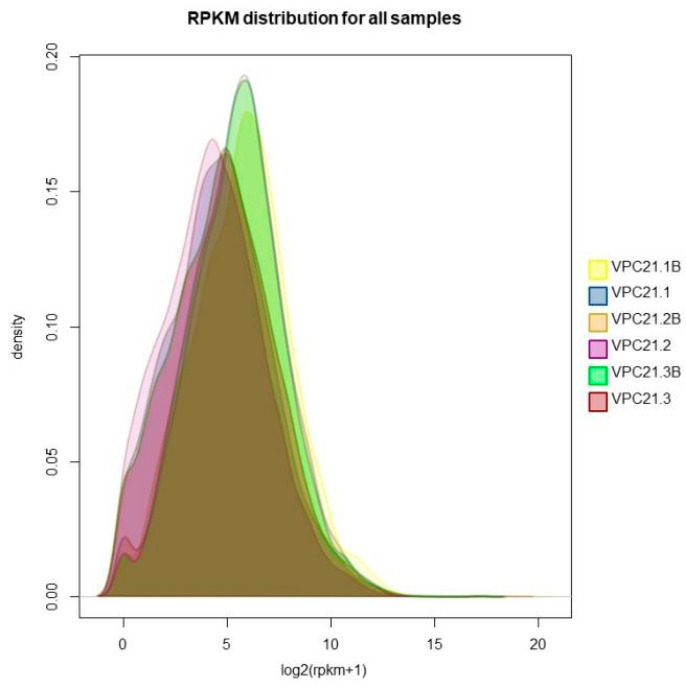
Density curve of the gene expression level of the *V. parahaemolyticus* biofilm and control group.

The read per million mapped reads (RPKM) is a method used to quantify the gene expression values using RNA-Seq technology. This method eliminates the influence of gene length and sequencing quantity on the calculated gene expression, allowing for a direct comparison of gene expression between different samples. Figure 4 shows the differences in the gene expression between the biofilm samples and the planktonic cell samples, with yellow, orange, and green representing the gene expression density of the VPC21 biofilm sample, and blue, purple, and red representing the gene expression density of the VPC21 planktonic cell sample. The results indicate that there is a difference in the gene expression between the biofilms and the planktonic cells.

**Figure 5:**
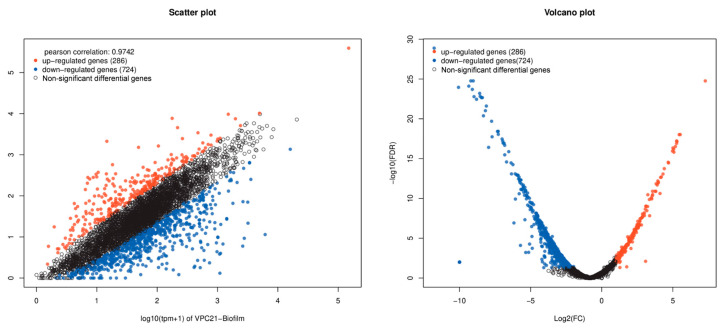
Visual scatter plot and volcano plot of *V. parahaemolyticus* transcriptome differential expression gene. Note: The red dots represent genes that are significantly upregulated, the blue dots represent genes that are significantly downregulated, and the black dots represent genes that are not significantly different.

Transcriptome gene expression differential analysis of the *V. parahaemolyticus* biofilm and planktonic cells were performed using the edgeR software (version 3.40.2). The analysis identified 984 differential genes, of which 724 antibiotic resistance associated genes were upregulated in biofilm producing *V. parahaemolyticus* when compared with the planktonic cells (Figure 5). The horizontal and vertical coordinates of the scatter plot represent the expression levels of the genes or transcripts in the two samples, respectively. The abscissa of volcano plots is the fold change value of the gene or transcript expression difference between them, and the ordinate is the statistical test value of the gene or transcript expression difference.

**Figure 6:**
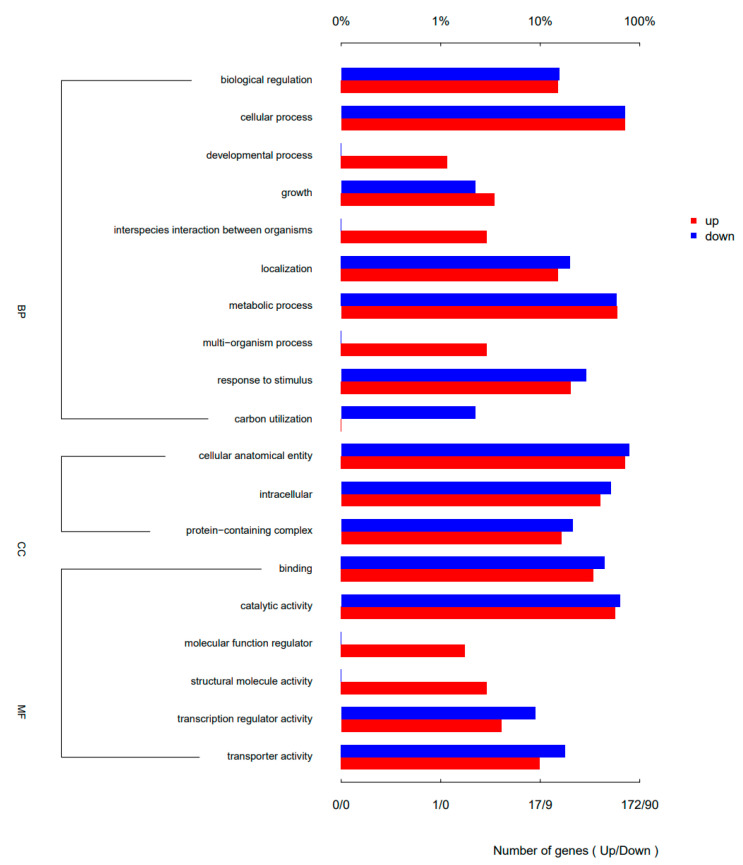
GO secondary annotation map of up- and downregulated differential genes. BP: Biological Process, CC: Cellular Component, MF: Molecular Function.

**Figure 7:**
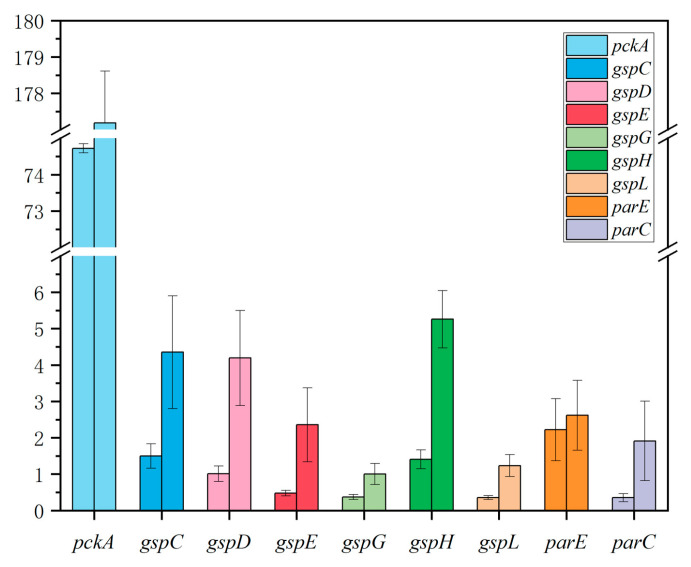
Quantitative reverse transcription polymerase chain reaction of upregulated genes. The left column represents the plankton cells sample, and the right column represents the biofilm sample.

A total of 172 antibiotic resistance associated genes were upregulated and 90 genes were downregulated in the biofilm annotated by GO annotations compared with the planktonic cells. The enrichment analysis results revealed that 19 GO terms were discovered in three categories: Biological Process, Cellular Component, and Molecular Function (Figure 6). There were more than 90 differential genes that were upregulated in the cellular process, metabolic process, cellular anatomical entity components, and catalytic activity. The differential expressions of genes related to anion transport and bacterial aggregation were particularly evident, with some material metabolism processes also showing signs of change. The upregulated genes were further verified by quantitative reverse transcription-polymerase chain reaction (q-PCR), and consistent results were obtained (Figure 7).

**Figure 8:**
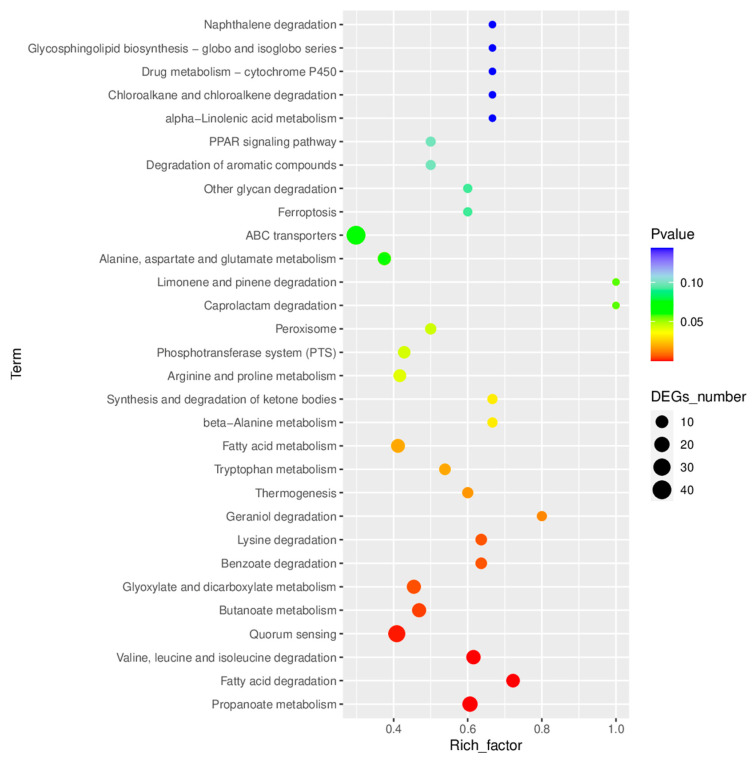
KEGG enrichment map of the differential expression genes.

KEGG enrichment analysis revealed that the expressions of 23 metabolic pathways including fatty acid, tryptophan, and arginine were upregulated after biofilm formation (Figure 8). Additionally, multiple functional pathways were significantly upregulated such as ABC transporters, phosphotransferase system-PTS, quorum sensing, DNA replication, bacterial secretion system, two-component system, bacterial chemotaxis, and flagellar assembly (Figure 8). In comparison, there were significantly fewer downregulated functional pathways including ascorbate and aldarate metabolism, monobactam biosynthesis, sphingolipid metabolism, apoptosis, etc.

## 3. Discussion

In this study, the ability of 32 strains of *V. parahaemolyticus* to form biofilm was determined by crystal violet staining and 29 strains demonstrated acceptable biofilm forming capabilities. Our analysis concluded that cells growing in biofilms were more resistant to antibiotics when compared to their planktonic living counterparts. On this basis, we posit a possible mechanism of antibiotic resistance of the *V. parahaemolyticus* biofilm to AGAs, which will be derived from observations of the structural properties of biofilms, the composition of extracellular polymeric substances, and transcriptomic sequencing.

### 3.1. “Enclosure” Type Resistance Mechanism of VP Biofilm against AGAs

Biofilms act as a barrier to reduce microbial cell susceptibility to antibiotics [26]. The antibiotic resistance of biofilm forming *Vibrio cholerae* type 0139 was shown to be significantly higher than that of planktonic cells to ampicillin, doxycycline, ciprofloxacin, erythromycin, and ceftriaxone (*p* < 0.05) [27]. Yonezawa et al. demonstrated that *Helicobacter pylori* significantly reduced the susceptibility to karaomycin after biofilm formation [28]. Garousi also showed that high numbers of isolates were able to form a biofilm, which is one of the contributing factors to antibiotic resistance [29]. Previously, Fauzia et al. pointed out that antibiotic resistance, shown by the minimum biofilm eradication concentration (MBEC) measurement, was higher in the prolific biofilm forming group of isolates for all antibiotics in comparison to isolates that were not prolific [30]. Many studies have shown that the formation of biofilm can significantly enhance the antibiotic resistance of microbial cells, but the mechanism promoting this ability is not explicitly known. In this study, the structural characteristics of a *V. parahaemolyticus* biofilm, the amount of biomass, average thickness, and biofilm roughness were measured and correlations between these structural characteristics and antibiotic resistance were quantified. The results show that the biomass of *V. parahaemolyticus* biofilm and the BIC of levofloxacin and gentamicin were linearly correlated (*p* < 0.05), and the correlation was stronger with amikacin (*p* < 0.01) (Table 7). Therefore, the antibiotic resistance of bacteria is enhanced by increased biofilm biomass. In addition, the average thickness of the *V. parahaemolyticus* biofilm was significantly correlated with the BIC for amikacin (*p* < 0.05). Cordeiro et al. demonstrated the enhanced antibiotic resistance of microbial cells after the treatment of *Candida albicans* with the β-lactam antibiotics cefepime and amoxicillin, and concluded that antibiotics stimulated biomass production and increased the biological volume and average thickness of these structures [31].

### 3.2. “Neutralization” Type Resistance Mechanism of VP Biofilm against AGAs

The EPSs of the biofilms, which include eDNA, exopolysaccharides, extracellular proteins, etc., act as a first line of defense against antibiotics by minimizing the concentration of antibiotics in the biofilm [32]. The EPSs thus shield bacteria present in the biofilm from antibiotics, while planktonic cells do not receive this protection [33]. Extracellular proteins [34] and eDNA [35] play critical roles in biofilm formation, and eDNA is an anionic macromolecule that can chelate cations [36]. Therefore, DNase I and proteinase K were used to reduce these matrix components of the *V. parahaemolyticus* biofilm and the antibiotic resistance of cells was measured in this depleted environment. It was found that the BIC of AGAs was lowered after enzymatic hydrolysis, showing that cells in the biofilms became more susceptible (Figure 3). Related studies have shown that the cell surface charge in biofilms undergo significant changes after treatment with amoxicillin and gentamicin [25]. It shows that biofilms reduce the effects of antibiotics through the reaction of anionic EPSs in the matrix and cationic AGAs, thus increasing the survivability of the cells in the biofilm. Similarly, Tseng et al. reported the inhibition of tobramycin permeation into the biofilm through the interaction of the negatively-charged EPSs with positively charged tobramycin [37]. In addition, evidence was provided that adding exogenous DNA can increase the tolerance of flow chamber-grown *Pseudomonas aeruginosa* wild-type biofilm to tobramycin treatments [38], Brown et al. also obtained similar conclusions by treating eDNA with DNase I [39]. The combined use of antibiotics with proteinase K by Shukla et al. significantly enhanced the inhibitory effect of antibiotics on *S. aureus* biofilm, which is consistent with the results of this study [40]. Furthermore, EPSs in *Pseudomonas aeruginosa* have also been shown to reduce the penetration of antibiotics through electrostatic interaction with antibiotics [41,42]. However, the BIC value of *V. parahaemolyticus* to antibiotics did not decrease after treatment with a higher concentration of enzymes, and we speculate that there is a threshold effect.

### 3.3. “Regulatory” Type Resistance Mechanism of VP Biofilm against AGAs

By sequencing the transcriptomes of cells growing in biofilms and planktonic cells, it was found that comparatively, the metabolic processes relating to anion transport and bacterial aggregation were enhanced in the biofilm cells. These results were observed via the differential expression of genes using GO annotation and enrichment analysis. A total of 724 antibiotic resistance associated genes were upregulated among 984 differential genes in biofilm producing *V. parahaemolyticus* (Figure 5). The ABC transport system, phosphotransferase system (PTS), quorum sensing, DNA replication, bacterial secretion system, two-component system, bacterial chemotaxis, flagellar assembly, and other functional pathways were significantly upregulated in the biofilm producing *V. parahaemolyticus* by KEGG enrichment analysis (Figure 8). ABC transporters that function as workers, in which they couple ATP-Mg binding, ATP hydrolysis, and ADP/phosphate release into the transmembrane transport of proteins, fats, polysaccharides, etc., by means of mechanical force [43]. In addition, ABC transporters can perform DNA repair and regulate gene expression [44]. It can be seen that the ABC transport system in the biofilm can enhance antibiotic resistance by facilitating the exchange of substances and the transfer of bacterial resistance. Li et al. showed that peptide RP557 may inhibit biofilm formation by downregulating nitrogen and fatty acid metabolism as well as peptidoglycan biosynthesis [45]. Zhao et al. reported that the expression level of *napA* gene was significantly upregulated after *Helicobacter pylori* biofilm formation, and the expression of *napA* in biofilm-forming cells of both wild-type and H57 strains was upregulated by 0.4 times compared with the planktonic cells [46].

## 4. Materials and Methods

### 4.1. Bacteria Solution Preparation

The 32 *V. parahaemolyticus* strains used in this study were provided by the Laboratory of Quality & Safety Risk Assessment for Aquatic Product on Storage and Preservation (Shanghai), Ministry of Agriculture and Rural Affairs. VPC16–VPC35 are clinical isolates from clinical diarrhea samples; VPD14–VPD61 and VPR103–VPR110 are from aquatic products and carry the *tdh* and *trh* virulence genes, respectively. The strains were streaked from a −80 °C stock onto TCBS agar (Beijing Land Bridge Technology Co., Ltd., Beijing, China), and incubated statically for 12–18 h at 37 °C. The independent single colony in the TCBS PETRI dish was selected and inoculated in an 8 mL TSB (Beijing Land Bridge Technology Co. Ltd., Beijing, China) tube containing 3% NaCl, then grown at 37 °C overnight with shaking at 200 rpm. The precipitation was separated from cultures by centrifugation at 3000 rpm for 10 min at room temperature. Then, the bacterial solution was thoroughly mixed by adding 0.85% NaCl to adjust the concentration to 9 Log CFU/mL.

### 4.2. Preparation of Antibiotic Sensitive Testing Plate (96-Well Plate)

According to the recommendations of the Clinical and Laboratory Standards Institute (CLSI, 2018), we used the following eight antibiotics: ampicillin (AMP), cefepime (CFPM), ceftazidime (CAZ), amikacin (AK), gentamicin (CN), tetracycline (TE) ciprofloxacin (CIP), and levofloxacin (LEV) (Sigma Aldrich Trading Co., Ltd., Shanghai, China). We prepared a 2048 μg/mL antibiotic stock solution aliquot and stored it at −20 °C. One milliliter antibiotic mother solution was mixed with 3 mL MHB (Thermo Fisher Technology China Co., Ltd., Shanghai, China) culture medium, and the same amount of MHB was added for 2-fold dilution to obtain 256 μg/mL of antibiotic solution. MHB (180 μL) was added to sterile 96-well polystyrene microtiter plates (Corning Management Co., Ltd., Shanghai, China), 180 μL of the 256 μg/mL antibiotic solution was added in the first column and were fully sucked, 180 μL of the mixed solution was aspirated in the first column into the second column, and then repeated. After mixing in the eleventh column, 180 μL of the solution was aspirated and discarded. The twelfth column served as a blank control. Varying concentrations of antibiotics were cryopreserved immediately.

### 4.3. Determination of Planktonic Cells Minimum Inhibitory Concentration (MIC)

We diluted the bacterial solution concentration to one tenth of the original with 0.85% normal saline and operated three times to an ultimate density of approximately 6 Log CFU/mL. A total of 20 μL bacterial solution was added to each well in the antibiotic sensitive testing plate, then the sample was mixed, and the OD_600_ was unchanged. After 24 h of growth at 37 °C, the MIC value was obtained by measuring the OD_600_ (Bio-Rad xMark plate reader) and recording the experimental results. The MIC was defined as the lowest concentration of antibiotic where no visual growth was observed.

### 4.4. Determination of Biofilm Minimum Inhibitory Concentration (BIC)

The minimum inhibitory concentration of biofilms (BIC) was determined by using a protocol given in Parker et al. [47]. Briefly, we added 100 μL of 6 Log CFU/mL bacterial solution into a 96-well plate with 100 μL TSB per well, and an inoculation needle was inserted on which the cells formed a biofilm (Thermo Fisher Technology China Co., Ltd., Shanghai, China). The 96-well plate containing the inoculation needle was incubated at 37 °C with shaking at 110 rpm for 24 h. The inoculation needle was removed and washed with 0.1 M PBS after the culture. These were later placed into a fully dissolved antibiotic sensitive plate and cultured at 37 °C for 24 h. The BIC value can be measured by measuring the OD_600_ and recording the experimental results.

### 4.5. Determination of Biofilm Biomass

The biofilm biomass was determined according to the reported method [48,49]. Briefly, for biofilm formation, the bacteria solution was diluted 1000 times with TSB containing 3% NaCl, and 1 mL bacterial solution was added into the well plate and cultured at 37 °C for 24 h. Biofilms were gently washed with PBS (Sangong Bioengineering Co., Ltd., Shanghai, China) later, three times, and were fixed for 10 min at 50 °C. Then, the biofilms were stained with 1 mL 0.1% (wt/vol) crystal violet (Sangong Bioengineering Co., Ltd., Shanghai, China) for 30 min, and washed with PBS three times. Subsequently the biofilms were dissolved with 1 mL ethanol (95%) (Sinopharm Chemical Reagents Co., Ltd., Shanghai, China) for 30 min. The optical density after the solution was completely dissolved was measured at the wavelength of 600 nm (OD_600_). Six parallel directions were performed for each experiment.

The OD_600_ value of each biofilm was compared with the ODc value of the negative control, and the biofilm formation ability was divided into four grades: If OD_600_ > 4ODc, the biofilm formation ability was rated as “strong”; the ability to form a biofilm was rated as “moderate” if 2ODc < OD_600_ ≤ 4ODc; the ability to form a biofilm was rated as “weak” if ODc < OD_600_ < 2ODc; if OD_600_ ≤ ODc, there was no biofilm formation ability. The raw data for this experiment are available in Table S1.

### 4.6. Confocal Laser Scanning Microscope (CLSM)

An analysis of the biofilms via confocal laser scanning microscopy followed the method used in Michler-Kozma [50]. Briefly, the inoculation solution was diluted 1000 times with TSB containing 3% NaCl, bacterial solution was added to a 24-well plate with 14 glass pieces in diameter and incubated at 37 °C for 24 h to cultivate biofilms. the planktonic cells were gently sucked away and the plates were washed three times with PBS. Subsequently, the sample was fixed in 4% glutaraldehyde at 4 °C for 30 min (Shanghai Yuanye Biotechnology Co., Ltd., Shanghai, China) and removed the residual fixative with PBS. Then, the fixed biofilm was dyed with SYBR Green I dye (Beijing Solebo Technology Co., Ltd., Beijing, China) at room temperature and washed with PBS 30 min later. The sample was placed on a glass slide and analyzed with a confocal laser scanning microscope (LSM710, Carl Zeiss, Jena, Germany). The excitation wavelength of the SYBR Green I fluorescence was 488 nm and the emission wavelength was 500–550 nm when observed with a 20-fold objective lens. Each slide was scanned three times at random locations, and the biovolume (BV), average thickness (AT), and biofilm roughness (BR) components were quantified via digital image analysis using ISA-2, which was provided by Professor Haluk Beyenal of Montana State University.

### 4.7. Enzyme Treatment of Biofilm (DNase I Enzyme and Protease K)

One mg/mL DNase I and protease K solution were added to a 96-well plate together with bacterial solution and liquid culture medium according to different concentration ratios, and then covered with a polystyrene micro titration plate (Microplate inoculation needle) (Thermo Fisher Technology China Co., Ltd., Shanghai, China) to make the biofilm form on the inoculation needle. The 96-well plates inserted with the inoculation needle were cultured at 110 rpm and 37 °C for 24 h. Then, we removed the inoculation needle and cleaned it with 0.1 M PBS three times. Next, we placed them into a fully dissolved antibiotic sensitive plate and cultured them at 37 °C for 24 h. The BIC value can be measured by measuring the OD_600_ and recording the experimental results. Samples were taken without the enzyme.

### 4.8. RNA-Sequencing

Biofilm and planktonic cells of VPC21 were selected and exposed to the antibiotic environment. The total bacterial RNA was extracted using the TRIzol^®^ reagent (Invitrogen, Waltham, MA, USA) according to the manufacturer’s instructions, and genomic DNA was removed using DNase I. The quality and quantity of the RNA were analyzed using the 2100 Bioanalyzer system and NanoDrop-2000 system, respectively. High-quality RNA samples (OD260/280 = 1.8–2.2, OD260/230 ≥ 2.0, RIN ≥ 6.5, 28 s:18S ≥ 1.0, >10 μg) were selected to construct the sequencing library. RNA extraction and follow-up work were completed by Shanghai Yunzhuo Biotechnology Co., Ltd., Shanghai, China. The RNA-Seq strand-specific library was prepared using 5 μg total RNA according to the Illumina TruSeq RNA Sample Preparation Kit (San Diego, CA, USA). The rRNA was then removed with a RIBO-Zero Magnetic Kit (Epicenter, Madison, WI, USA), and the buffer was used for fragmentation. According to the Illumina protocol, cDNA synthesis, terminal repair, A-base addition, and ligation of the Illumina Index connector were performed. Then, library sizes of 200–300 bp cDNA target fragments were selected on 2% agarose, and 15 PCR cycles were amplified using the Phusion DNA polymerase. After quantification by TBS380, the paired terminal library was sequenced by Illumina NovaSeq 6000 sequencing. The sequence information has been uploaded to NCBI, and the sequence number is SRP376054.

In order to determine the differentially expressed genes of the biofilm and planktonic cells, the expression level of each transcript was calculated using the fragments per kilobase of read per million mapped reads (RPKM) method [51]. Differential gene expression was analyzed by https://bioconductor.org/packages/release/bioc/html/edgeR.html (accessed on 2 June 2022) [52]. We selected the DEG (differentially expressed genes) between the two samples using the following criteria: (i) the logarithm of the fold change is greater than 2; (ii) the false discovery rate (FDR) should be less than 0.05. To understand the function of differentially expressed genes, GO (Gene Ontology) and KEGG (Kyoto Encyclopedia of Genes and Genomes) enrichment analyses were performed.

### 4.9. Quantitative Reverse Transcription-Polymerase Chain Reaction (q-PCR)

The quantitative reverse transcriptase-polymerase chain reaction procedure was adjusted based on the existing literature and forwarded to the Shanghai Yunzhuo Technology Co., Ltd. for finalization [53]. Refer to Table S2 for details on the primers used.

### 4.10. Statistical Analysis

Statistical analysis and graphs were generated by SPSS26.0 statistical software (SPSS Statistics, Inc., Chicago, IL, USA), Spearman rank correlation analysis was performed, and a *p* < 0.05 was considered as a statistically significant level. Origin 2021 (OriginLab Corporation, Northampton, MA, USA) was used for mapping, and the data are represented as the mean ± SEM (standard error of mean). The structural parameters of the biofilm (biological volume, average thickness, roughness coefficient) were studied by using the structural analysis software ISA-2 [54].

## 5. Conclusions

**Figure 9:**
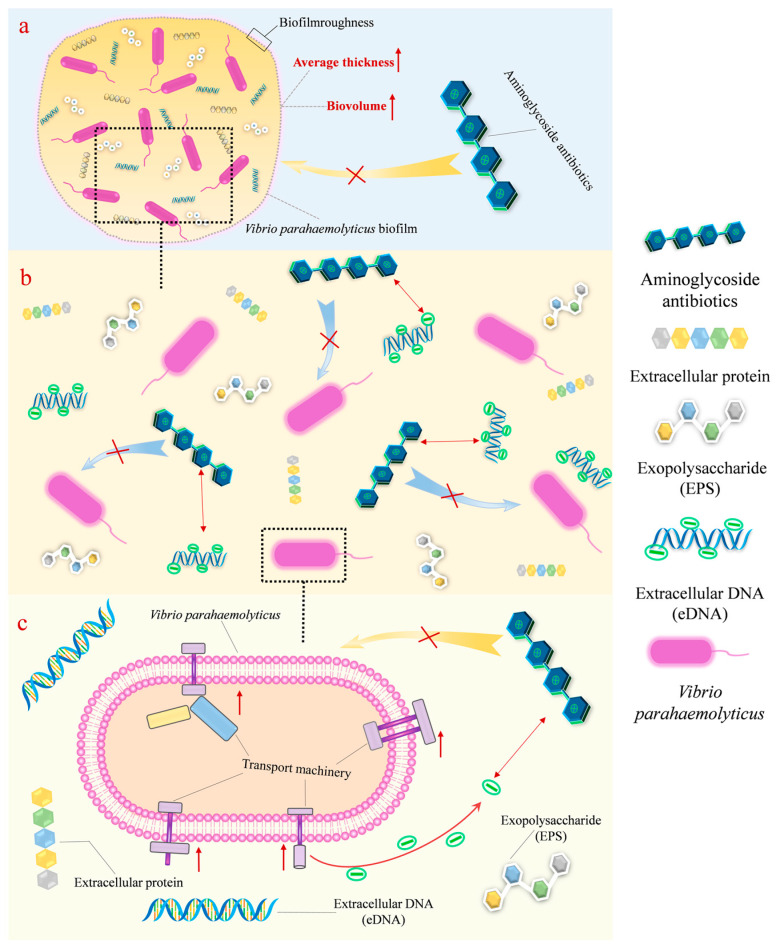
New mechanisms of biofilm resistance to aminoglycoside antibiotics. (**a**) Enclosure mechanism; (**b**) neutralization mechanism; (**c**) regulatory mechanism.

A possible mechanistic explanation of the antimicrobial resistance of *V. parahaemolyticus* biofilm cells to AGAs was shown in this study (Figure 9). These mechanisms were: (i) enclosure mechanism: whereby the generation of AGA resistance was observed as a result of increasing the biofilm biomass and average thickness; (ii) neutralization mechanism: reduction of antibiotic levels in the *V. parahaemolyticus* biofilm, which occurred via the binding of anionic EPSs in the biofilm to cationic antibiotics; (iii) regulatory mechanism: upregulation of antibiotic resistance associated genes in biofilm producing *V. parahaemolyticus* compared with planktonic cells. The three mechanistic strategies of developing resistance contribute to a better understanding of biofilm antibiotic resistance and provides a route to the effective treatment of bacterial infections. Selective and judicious use of new antibiotics is needed to win the battle against infectious diseases.

## Figures and Tables

**Table 1:** *V. parahaemolyticus* and biofilm-forming ability.

	Biofilm-Forming Ability					Biofilm-Forming Ability			
	Strong (+++)	Moderate (++)	Weak (+)	No Biofilm (−)		Strong (+++)	Moderate (++)	Weak (+)	No Biofilm (−)
VPC16			+		VPD8				**−**
VPC17	+++				VPD14			+	
VPC18			+		VPD18	+++			
VPC19			+		VPD33			+	
VPC20	+++				VPD34			+	
VPC21	+++				VPD57			+	
VPC22	+++				VPD58			+	
VPC25		++			VPD61	+++			
VPC26		++			VPR101		++		
VPC27			+		VPR103		++		
VPC28	+++				VPR104	+++			
VPC29	+++				VPR105			+	
VPC32	+++				VPR106				**−**
VPC33			+		VPR108			+	
VPC34		++			VPR110			+	
VPC35	+++				VPR111				**−**

**−**: The strain has no biofilm-forming ability, +: The biofilm-forming ability of the strain is “weak”, ++: The biofilm-forming ability of the strain is “moderate”, +++: The biofilm-forming ability of the strain is “strong”.

**Table 2:** Minimum inhibitory concentration (MIC) of *V. parahaemolyticus* planktonic cells against the antibiotics.

	AMP	CFPM	CAZ	AK	CN	TE	CIP	LEV
VPC16	>128	0.25	0.25	8	4	0.5	0.125	0.5
VPC17	128	0.5	0.25	16	2	0.5	0.5	0.5
VPC18	>128	0.5	0.25	16	4	0.5	0.25	0.25
VPC19	>128	0.5	0.25	16	4	0.5	0.25	0.25
VPC20	>128	0.5	0.5	8	4	0.5	0.25	0.25
VPC21	128	0.25	0.25	8	2	0.5	0.125	0.125
VPC22	>128	0.5	0.25	16	4	0.5	0.25	0.125
VPC25	>128	0.5	0.25	16	2	0.5	0.25	0.125
VPC26	128	0.5	0.25	16	8	0.25	0.25	0.125
VPC27	32	0.5	0.25	8	4	0.25	0.125	0.125
VPC28	>128	0.5	0.5	16	8	0.25	0.25	0.125
VPC29	128	0.5	0.5	16	8	0.25	0.25	0.25
VPC32	64	0.5	0.25	16	8	0.5	0.25	0.125
VPC33	>128	0.25	0.25	16	4	0.25	0.25	0.125
VPC34	16	0.5	0.25	8	4	0.25	0.125	0.125
VPC35	64	0.5	0.25	16	8	0.5	0.25	0.125
VPD8	>128	0.125	0.5	16	8	0.25	0.25	0.25
VPD14	128	1	0.25	16	8	0.5	0.25	0.25
VPD18	>128	0.5	8	16	4	0.5	16	8
VPD33	>128	0.125	0.25	16	8	0.25	0.25	0.25
VPD34	>128	1	0.5	16	8	0.5	0.25	0.25
VPD57	128	0.5	0.125	16	4	0.5	0.5	0.25
VPD58	128	0.25	0.25	8	4	0.25	8	0.25
VPD61	>128	1	0.5	16	8	0.5	0.25	0.25
VPR101	128	0.125	0.5	32	4	0.25	0.25	0.25
VPR103	>128	0.5	0.5	16	8	0.5	0.25	0.125
VPR104	64	0.5	0.25	16	4	0.5	0.125	0.25
VPR105	>128	0.5	0.25	16	4	0.25	0.25	0.125
VPR106	>128	0.5	0.25	16	8	0.25	0.25	0.25
VPR108	>128	0.5	0.25	16	4	0.5	0.25	0.25
VPR110	>128	0.5	0.5	16	8	0.5	0.25	0.25
VPR111	16	0.25	0.125	8	4	0.25	0.125	0.125

AMP: ampicillin, CFPM: cefepime, CAZ: ceftazidime, AK: amikacin, CN: gentamicin, TE: tetracycline, CIP: ciprofloxacin, LEV: levofloxacin.

**Table 3:** Linear correlation between the antibiotic resistance of planktonic cells and the biofilm formation ability.

	AMP	CFPM	CAZ	AK	CN	TE	CIP	LEV
SCC	−0.016	0.169	0.153	0.064	−0.012	0.130	0.002	−0.117
*p*-value	0.935	0.38	0.428	0.741	0.95	0.501	0.990	0.547

AMP: ampicillin, CFPM: cefepime, CAZ: ceftazidime, AK: amikacin, CN: gentamicin, TE: tetracycline, CIP: ciprofloxacin, LEV: levofloxacin.

**Table 4:** The minimum inhibitory concentration of biofilm (BIC) against antibiotics.

	AMP	CFPM	CAZ	AK	CN	TE	CIP	LEV
VPC16	64	2	0.5	8	8	0.5	0.25	0.25
VPC17	>128	0.5	2	32	8	0.5	0.5	0.5
VPC18	>128	0.25	0.25	16	8	0.25	1	0.125
VPC19	>128	0.5	0.5	32	4	0.5	0.25	0.25
VPC20	>128	0.5	0.25	32	4	0.25	0.25	0.5
VPC21	>128	0.25	1	32	16	0.25	0.5	0.25
VPC22	>128	0.5	0.25	32	8	0.5	0.25	0.5
VPC25	>128	0.25	0.25	32	2	0	0.25	0.125
VPC26	>128	8	8	32	16	1	1	0.5
VPC27	64	0.5	0.25	16	8	0.25	0.25	0.25
VPC28	>128	0.5	0.5	64	16	1	2	2
VPC29	128	0.25	0.5	32	16	0.25	0.25	0.25
VPC32	64	1	4	32	16	1	2	2
VPC33	>128	8	2	32	8	1	0.5	0.25
VPC34	64	0.5	0.25	16	8	1	0.25	0.25
VPC35	64	1	4	64	16	1	0.25	0.5
VPD14	128	4	4	32	8	0.5	0.25	1
VPD18	>128	0.5	8	32	8	0.5	8	8
VPD33	>128	0	8	16	8	0.25	0.25	0.125
VPD34	>128	1	2	16	8	0.5	1	0.25
VPD57	128	1	1	16	8	1	0.5	0.125
VPD58	128	0	1	8	4	0.125	0.125	0.125
VPD61	>128	1	1	32	8	0.25	0.25	0.125
VPR101	>128	0	2	32	8	0.25	0.25	0.25
VPR103	>128	16	2	32	16	16	2	1
VPR104	64	8	8	32	8	4	1	1
VPR105	64	2	4	16	8	2	1	0.5
VPR108	64	8	8	16	16	1	0.5	0.125
VPR110	128	8	8	64	16	4	0.5	0.25

AMP: ampicillin, CFPM: cefepime, CAZ: ceftazidime, AK: amikacin, CN: gentamicin, TE: tetracycline, CIP: ciprofloxacin, LEV: levofloxacin.

**Table 5:** Correlation between the antibiotic resistance of biofilm (BIC) and the biofilm formation ability.

	AMP	CFPM	CAZ	AK	CN	TE	CIP	LEV
SCC	0.231	−0.271	−0.081	0.530 *	−0.083	−0.254	0.018	0.352
*p*-value	0.229	0.154	0.677	0.003	0.668	0.184	0.927	0.061

* *p*-value < 0.01 AMP: ampicillin, CFPM: cefepime, CAZ: ceftazidime, AK: amikacin, CN: gentamicin, TE: tetracycline, CIP: ciprofloxacin, LEV: levofloxacin.

**Table 6:** Biofilm structure parameters of *V. parahaemolyticus*.

Strains	BV (×10^5^ µm^3^)	AT (µm)	BR
VPC16	3.67 ± 0.51	3.10 ± 0.79	1.64 ± 0.04
VPC17	12.68 ± 1.79	12.35 ± 1.78	1.62 ± 0.24
VPC18	1.19 ± 0.52	1.25 ± 0.49	1.88 ± 0.05
VPC19	1.08 ± 0.32	0.91 ± 0.15	1.89 ± 0.04
VPC20	11.18 ± 0.47	11.16 ± 0.41	1.86 ± 0.06
VPC21	17.18 ± 6.15	10.15 ± 9.84	1.16 ± 0.73
VPC22	15.32 ± 4.90	17.78 ± 4.90	1.10 ± 0.32
VPC25	10.38 ± 1.13	10.00 ± 0.83	1.00 ± 0.04
VPC26	2.13 ± 0.04	0.15 ± 0.07	1.98 ± 0.01
VPC27	3.45 ± 1.91	3.54 ± 2.77	1.65 ± 0.91
VPC28	11.97 ± 1.01	12.64 ± 1.44	1.67 ± 0.15
VPC29	13.69 ± 1.56	3.63 ± 1.29	1.55 ± 0.17
VPC32	14.79 ± 0.12	0.88 ± 0.12	1.91 ± 0.01
VPC33	11.35 ± 4.68	11.22 ± 2.90	0.89 ± 0.15
VPC34	8.72 ± 5.25	8.02 ± 5.63	1.26 ± 0.42
VPC35	22.04 ± 0.44	12.00 ± 0.31	1.79 ± 0.04
VPD14	1.65 ± 0.56	1.58 ± 0.68	1.80 ± 0.07
VPD18	17.40 ± 2.32	18.62 ± 2.01	0.96 ± 0.16
VPD33	1.47 ± 0.94	1.39 ± 0.94	1.86 ± 0.10
VPD34	1.68 ± 1.07	1.71 ± 1.05	1.83 ± 0.09
VPD57	1.48 ± 0.90	1.55 ± 0.67	1.81 ± 0.1
VPD58	4.82 ± 3.83	4.71 ± 4.08	1.48 ± 0.38
VPD61	17.24 ± 2.03	13.90 ± 2.31	0.78 ± 0.04
VPR101	16.05 ± 3.42	15.95 ± 2.59	1.27 ± 0.37
VPR103	14.64 ± 3.37	15.29 ± 3.06	1.43 ± 0.27
VPR104	17.38 ± 3.05	17.71 ± 2.04	1.18 ± 0.24
VPR105	0.74 ± 0.51	0.99 ± 0.50	1.88 ± 0.06
VPR108	3.39 ± 0.31	3.06 ± 0.52	1.63 ± 0.04
VPR110	17.22 ± 6.36	17.05 ± 5.65	1.29 ± 0.52

BV: biovolume, AT: average thickness, BR: biofilm roughness.

**Table 7:** Correlation between the antibiotic resistance of the biofilm and biofilm structure.

	BV (×10^5^ µm^3^)		AT (µm)		BR	
	SCC	*p*-Value	SCC	*p*-Value	SCC	*p*-Value
Ampicillins	0.018	0.924	0.101	0.601	−0.143	0.459
Cefepime	0.016	0.933	0.11	0.957	0.053	0.784
Ceftazidime	0.066	0.733	0.021	0.914	0.094	0.628
Amikacin	0.813 **	0.000	0.642 **	0.000	0.029	0.883
Gentamicin	0.411 *	0.027	0.130	0.501	−0.283	0.137
Tetracycline	0.136	0.483	0.088	0.649	0.115	0.553
Ciprofloxacin	0.117	0.547	−0.039	0.841	0.115	0.422
Levofloxacin	0.430 *	0.03	0.272	0.153	0.096	0.62

SCC, Spearman correlation coefficient, * *p*-value < 0.05; ** *p*-value < 0.01. BV, biovolume; AT, average thickness; BR, biofilm roughness.

## Data Availability

The data presented in this study are available in the manuscript and the Supplemental Materials. Additional information may be requested from the corresponding author.

## Supplementary Materials

The following supporting information can be downloaded at: https://www.mdpi.com/article/10.3390/antibiotics12040638/s1, Table S1: The OD_600_ value of *V. parahaemolyticus* biofilm; Table S2: Details of primer pairs.
