# Supplementary material for: Discovery of Novel Resistance Mechanisms of Vibrio parahaemolyticus Biofilm against Aminoglycoside Antibiotics
Source: Antibiotics (Basel). 2023 Mar 24;12(4):638. doi: 10.3390/antibiotics12040638 (PMC10135303; doi:10.3390/antibiotics12040638)
Supplement: Supplementary file 1 [file antibiotics-12-00638-s001.zip › antibiotics-2133416-supplementary.pdf]

antibiotics

MDPI

Supplementary

Discovery of Novel Resistance Mechanisms of *Vibrio parahaemolyticus* Biofilm against Aminoglycoside Antibiotics

Cuifang Tian [1,†], Mengqi Yuan [1,†], Qian Tao [1], Tianming Xu [1], Jing Liu [1], Zhenhua Huang [1], Qian Wu [1], Yingjie Pan [1,2,3], Yong Zhao [1,2,3,*] and Zhaohuan Zhang [1,*]

Table S1 The OD_{600} value of *V. parahaemolyticus* biofilm

	OD	OD_C	$2OD_C$	$4OD_C$
VPC16	0.36±0.08	0.136±0.02	0.272±0.02	0.544±0.02
VPC17	1.23±0.03	0.136±0.02	0.272±0.02	0.544±0.02
VPC18	0.70±0.13	0.136±0.02	0.272±0.02	0.544±0.02
VPC19	0.74±0.02	0.136±0.02	0.272±0.02	0.544±0.02
VPC20	1.14±0.02	0.136±0.02	0.272±0.02	0.544±0.02
VPC21	3.40±1.13	0.136±0.02	0.272±0.02	0.544±0.02
VPC22	0.75±0.11	0.136±0.02	0.272±0.02	0.544±0.02
VPC25	0.73±0.18	0.136±0.02	0.272±0.02	0.544±0.02
VPC26	0.95±0.44	0.136±0.02	0.272±0.02	0.544±0.02
VPC27	0.57±0.17	0.136±0.02	0.272±0.02	0.544±0.02
VPC28	1.25±0.28	0.136±0.02	0.272±0.02	0.544±0.02
VPC29	0.43±0.17	0.136±0.02	0.272±0.02	0.544±0.02
VPC32	1.04±0.17	0.136±0.02	0.272±0.02	0.544±0.02
VPC33	0.45±0.01	0.136±0.02	0.272±0.02	0.544±0.02
VPC34	0.71±0.03	0.136±0.02	0.272±0.02	0.544±0.02
VPC35	0.88±0.26	0.136±0.02	0.272±0.02	0.544±0.02
VPD8	0.35±0.08	0.193±0.01	0.386±0.01	0.772±0.01
VPD14	1.15±0.48	0.193±0.01	0.386±0.01	0.772±0.01
VPD18	0.85±0.14	0.193±0.01	0.386±0.01	0.772±0.01
VPD33	0.70±0.02	0.193±0.01	0.386±0.01	0.772±0.01
VPD34	0.51±0.01	0.193±0.01	0.386±0.01	0.772±0.01
VPD57	0.40±0.02	0.193±0.01	0.386±0.01	0.772±0.01
VPD58	0.39±0.04	0.193±0.01	0.386±0.01	0.772±0.01
VPD61	1.09±0.06	0.193±0.01	0.386±0.01	0.772±0.01
VPR101	0.96±0.03	0.193±0.01	0.386±0.01	0.772±0.01
VPR103	0.26±0.03	0.193±0.01	0.386±0.01	0.772±0.01
VPR104	0.43±0.22	0.193±0.01	0.386±0.01	0.772±0.01

VPR105	0.42±0.74	0.193±0.01	0.386±0.01	0.772±0.01
VPR106	0.37±0.29	0.193±0.01	0.386±0.01	0.772±0.01
VPR108	0.50±0.04	0.193±0.01	0.386±0.01	0.772±0.01
VPR110	0.49±0.07	0.193±0.01	0.386±0.01	0.772±0.01
VPR111	0.23±0.01	0.193±0.01	0.386±0.01	0.772±0.01

Notes: The values are mean value ± standard deviation.

Table S2 Details of primer pairs

**Target Genes**	**Primer Sequence**	**Expected Size (bp)**
pckA	TCGGTAGGACGAATGAACATG<br>TGACGGTTATTGTGGTGCTAA	108
gspC	GCAGGATTATCAAGCAGACC<br>TATCGCAATAAATAGGCAAGTAAG	102
gspD	GTGACTCAGATACTGGTGCTAT<br>ATACTTGGAGAAGATAGGATGTTTG	187
gspE	TACGCTCTGTTCTGACTGTAAAG<br>CTTAGACCATCACTGCGAATACTT	270
gspG	GGTGGTTACATCAAGCGTCTT<br>GAAGTCTTGCATGTTCCAGTTG	165
gspH	AACCACCGCAAGTCTTCATT<br>GCATTCGCATCCTCTTCCA	169
gspL	TGCGGGCGTTTTGTTAGTG<br>GCAGCAAGCCAAGACAACAT	228
parE	CGTTGAAGCGGGTCATATCTAT<br>ACCTAGCAGCATGTCCATCA	280
parC	GAGCGTGACCTAGTACCAAGT<br>GTGCGACTCAAGCGAGTAAC	204
